# Artificial intelligence for ophthalmic vessel segmentation: from multimodal imaging to clinical translation

**DOI:** 10.3389/fmed.2026.1912838

**Published:** 2026-10-19

**Authors:** Lingyan Wu, Jiahao Tang, Shanping Zhao

**Affiliations:** 1 Hangzhou Lin'an Traditional Chinese Medicine Hospital, Affiliated Hospital of Hangzhou City University, Hangzhou, China; 2 School of Ophthalmology and Optometry, Wenzhou Medical University, Wenzhou, China

**Keywords:** artificial intelligence, clinical translation, corneal neovascularization, ophthalmic imaging, optical coherence tomography angiography, retinal vessels, structural metrics, vessel segmentation

## Abstract

Artificial intelligence (AI)-based vessel segmentation converts color fundus photographs, scanning laser ophthalmoscopy (SLO), optical coherence tomography angiography (OCTA), structural OCT, and anterior-segment images into quantitative vascular biomarkers. Strong performance within individual datasets has not consistently yielded reliable clinical measurements because errors can arise during acquisition, anatomical preprocessing, vessel extraction, biomarker calculation, and deployment. This structured narrative review compares segmentation tasks involving retinal vascular trees, OCTA capillary plexuses, the structural choroid and choriocapillaris, macular neovascularization, corneal neovascularization, and conjunctival microvasculature. We assess modern architectures in relation to vessel geometry, severe foreground-background imbalance, scale variation, crossings, and susceptibility to artifacts. The methods considered include multiscale feature pyramids, deformable and directional operators, Transformers, state-space models, diffusion approaches, and foundation models. We organize the synthesis around five evaluation levels: pixel overlap, centerline recovery, vascular topology, biomarker agreement, and clinical actionability. Public fundus and OCTA datasets are catalogued with their acquisition and annotation characteristics, and gaps in public anterior-segment vessel benchmarks are discussed. Because devices, data splits, labels, and post-processing differ substantially, reported metrics are summarized without ranking studies against one another. The review distinguishes structural OCT choroidal vascularity index from OCTA choriocapillaris flow-deficit mapping, as well as true vessel segmentation from global hyperemia grading. Clinical translation will require ocular-magnification correction, quality control of retinal layers and projection artifacts, external testing across devices and populations, out-of-distribution rejection, DICOM/PACS interoperability, prospective impact studies, and regulation as software as a medical device. We conclude that clinical utility depends on an auditable pipeline that measures structural error, biomarker uncertainty, and failure states throughout the workflow.

## 1 Introduction

The ocular vasculature can be examined non-invasively and provides information about both ocular and systemic disease. Retinal caliber, branching geometry, tortuosity, arteriovenous ratio, fractal dimension, and vessel density have been studied in diabetic and hypertensive retinopathy, retinal vein occlusion (RVO), retinopathy of prematurity, age-related macular degeneration (AMD), high myopia, cardiovascular disease, and stroke (1–9). Reliable segmentation underpins these measurements and is a key analytical step in estimating anatomical and flow-related biomarkers.

The anatomical target and image formation process differ by modality. Color fundus photography shows a two-dimensional vascular tree in which arteries and veins overlap at crossings. SLO and ultra-widefield (UWF) imaging introduce different contrast mechanisms, fields of view, and patterns of peripheral distortion. OCTA derives motion contrast from repeated OCT scans and depicts superficial and deep capillary plexuses, although every en face slab depends on structural layer segmentation and artifact suppression. Structural OCT records tissue reflectivity for choroidal compartment analysis, whereas choriocapillaris OCTA measures flow signal near the device's resolution and sensitivity limits. Slit-lamp and ocular-surface imaging add the difficulties of curved, reflective, and mobile surfaces (10–14).

Existing reviews address retinal vessel segmentation, OCTA analysis, or disease-specific imaging biomarkers from different perspectives (15–18). Our contribution is an integrative, task-aware synthesis of the measurement chain, from acquisition and anatomical preprocessing to vessel segmentation, structural and biomarker validation, and deployment. It is not a new algorithm or a validated performance score. The central question is whether an apparently plausible mask preserves the measurement needed for a defined clinical use. A broken terminal capillary, an artery-vein identity switch, and a displaced OCTA slab illustrate different failure mechanisms that require different validation endpoints.

This review has five objectives: (i) to distinguish true ophthalmic vessel segmentation from tissue segmentation, flow-deficit mapping, and redness analysis; (ii) to relate vessel geometry to architecture and loss design; (iii) to compare evaluation at the pixel, centerline, graph, biomarker, and clinical levels; (iv) to catalogue multimodal datasets and representative quantitative results; and (v) to describe a practical route from technical performance to prospective validation, interoperability, quality control, and regulatory review. Figure 1 summarizes the resulting vessel-to-biomarker pipeline.

**Figure 1:**
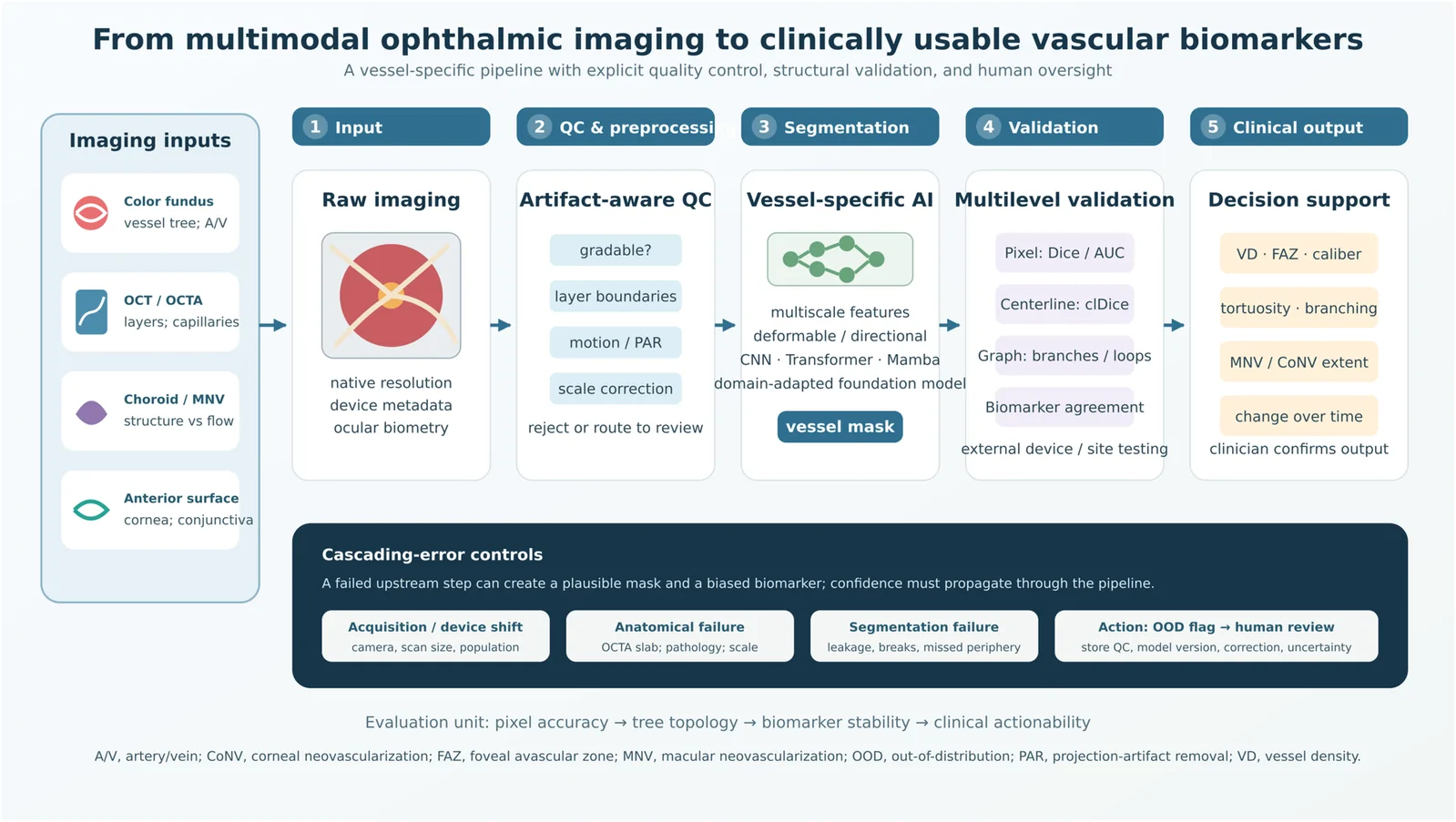
Conceptual schematic of multimodal ophthalmic vascular AI as an auditable measurement pipeline; the illustrated input is schematic, not a patient image or an experimental result. Raw color fundus or SLO images, OCT or OCTA, choroidal or MNV imaging, and anterior-segment images first undergo modality-specific quality control (QC). Preprocessing includes gradability assessment, verification of structural layers for OCTA, motion and projection-artifact removal (PAR), region definition, and correction of physical scale using device and ocular-biometry metadata. Vessel-specific models then generate feature representations and masks; the architecture families shown are examples rather than a prescribed model. Validation advances from pixel overlap to centerline continuity, graph structure, biomarker agreement, and external testing across devices and sites. Clinical outputs include vessel density (VD), foveal avascular zone (FAZ), caliber, tortuosity, branching, and the extent of macular or corneal neovascularization, with clinician confirmation. The lower band shows how errors can propagate through the pipeline and where out-of-distribution (OOD) rejection is required. A/V, artery/vein; CoNV, corneal neovascularization; MNV, macular neovascularization.

## 2 Literature identification and critical appraisal

This article is a structured narrative review rather than a systematic review or meta-analysis. It covers ophthalmic vessel segmentation and related measurement tasks across fundus photography, SLO/UWF, OCT/OCTA, and anterior-segment imaging. The final bibliography was assembled purposively and iteratively around modality, task, model, validation, and translation themes. Because contemporaneous search logs were not retained for the initial selection, that process is not presented as systematic or exhaustive. During revision, bibliographic identities were checked against original publications, database records, or official sources; reference verification was treated separately from literature discovery.

To evaluate reproducibility during revision, we conducted a retrospective coverage audit of the fixed 72-reference bibliography. Six PubMed/MEDLINE modules were executed on 13 September 2026, with publication dates limited through 31 August 2026; no language filter was applied. The first four modules combined terms for ophthalmic modalities and vascular targets with segmentation or quantification, image artifacts and domain shift, topology-aware or foundation-model methods, and narrative-review appraisal. They returned 14,850, 26,318, 1,558, and 3,972 records, respectively; their union contained 44,471 unique PubMed records and retrieved 56 of the 62 PubMed-indexed references. After gap analysis, two broader supplementary modules addressing ophthalmic imaging context and clinical AI translation returned 18,458 and 21,971 records. The union of all six modules contained 79,090 unique records and retrieved all 62 PubMed-indexed references. Query membership was tested by intersecting each complete query with the resolved PMID set; the full yield was not reconstructed from a capped export.

Two IEEE Xplore searches were run on the same date and returned 1,412 and 891 records; together they retrieved two non-PubMed conference papers. Five regulatory or interoperability documents were located on official FDA, EUR-Lex, European Commission, and DICOM websites. Three remaining engineering papers were verified by exact title and DOI or official proceedings but were not counted as unbiased database retrieval. Web of Science strategies were specified but not executed because subscription access was unavailable; no Web of Science hit counts are reported. Complete Boolean strings, query-level yields, and reference-level retrieval routes are provided in the accompanying search log.

Because the two broad PubMed modules were added after examination of the initial misses and the 79,090 records were not prospectively screened as a systematic-review corpus, these results are reported only as a high-sensitivity revision-stage coverage audit. They must not be interpreted as a reconstructed PRISMA flow or as evidence that the entire database yield was independently screened.

The synthesis distinguishes primary algorithmic and clinical studies, dataset/benchmark reports, methodological papers, contextual reviews, and regulatory or interoperability documents. Eligible sources were peer-reviewed primary studies, full conference papers, benchmark or dataset reports, clinically relevant reviews, and authoritative guidance or standards that defined a relevant imaging modality, vascular or related measurement target, validation setting, or translation requirement. Duplicate reports, abstracts lacking sufficient methods or results, disease-classification studies without a vessel, vascular, tissue, flow-deficit, or redness measurement, unrelated organ or lesion segmentation, and records whose bibliographic identity could not be verified were excluded. Candidate titles and abstracts were assessed for scope, available full texts were consulted where accessible, and the final citations were reconciled by DOI, PMID, exact title, and publisher or official record. This was not duplicate independent screening of the full database yield. Primary vessel-segmentation evidence requires a vascular target and an identifiable validation procedure; disease classification, global redness grading, and tissue segmentation are treated as adjacent tasks, not interchangeable segmentation outcomes. General methods are discussed where they inform vascular topology or model adaptation. The 72 references constitute a mixed bibliography across these evidence types, not 72 independently eligible segmentation studies. Quantitative results are presented descriptively without pooling because study populations, image units, data splits, reference labels, thresholds, post-processing, and metrics differ.

Critical appraisal focuses on data availability, patient-level separation, external site/device testing, artifacts and ungradable inputs, structural and biomarker outcomes, computational reporting, and the clinical reference standard. Missing or inaccessible information is treated as uncertainty, not proof of poor conduct. SANRA informs transparent reporting of this narrative review; it is not used as a risk-of-bias instrument for individual AI studies (19). No formal study-level summary score or independent reanalysis of model predictions is presented. The tables and discussion identify limitations of representative studies without implying that all sources underwent an identical full-text appraisal.

## 3 Modality-specific task definitions and failure modes

### 3.1 Color fundus photography, SLO, and the retinal vascular tree

Color fundus photography is the most established setting for retinal vessel segmentation. Large vessels are usually conspicuous, whereas low-contrast terminal branches, the optic-disc margin, central light reflexes, and lesion-adjacent vessels account for many of the clinically important errors. Because background occupies most of the image, a high overall accuracy can mask poor sensitivity to fine vessels. Classical matched and directional filters, morphological operators, and vesselness measures remain useful for enhancement and centerline extraction, although supervised benchmarks are now dominated by deep encoder-decoder networks (1, 17, 18, 20–22).

Pathology changes vessel appearance and can alter the effective task. Hemorrhages may resemble dark vessel segments, while exudates and cotton-wool spots interrupt contrast boundaries. Intraretinal microvascular abnormalities (IRMA) and neovascularization at the disc or elsewhere (NVD/NVE) form tortuous networks that a model may discard as noise. RVO changes caliber and local contrast, and laser scars or media opacity introduce nonvascular edges. Evaluation on predominantly healthy benchmark images will not reveal these errors. Robust studies should instead report lesion-stratified performance and separate false-positive lesion borders from missed pathological vessels. Multi-task learning with lesion masks, uncertainty labels for ambiguous pixels, hard-negative sampling, and topology-aware refinement may help, but these approaches depend on disease-rich annotations.

SLO and UWF images should be evaluated separately from standard flash photography. SLO produces contrast through point scanning and confocal detection. UWF systems cover up to approximately 200 degrees but introduce peripheral stretching, eyelash and eyelid artifacts, and nonuniform illumination. IOSTAR is consequently an SLO benchmark rather than a conventional color-fundus dataset. PRIME-FP20 illustrates both the annotation burden and the modality gap in UWF photography, using paired UWF fluorescein angiography to support weak supervision (23).

### 3.2 Artery-vein differentiation is a graph-labeling problem

Artery-vein (A/V) analysis is a graph-labeling task, not simply an extra class in a binary vessel mask. Color and caliber are unreliable cues in small branches, and arteries and veins overlap at crossings without changing identity. A valid system must distinguish a crossing from a bifurcation, maintain labels across short discontinuities, and propagate identity from the optic disc along the vascular tree. One error in a high-order branch can bias downstream arteriolar-to-venular ratio and caliber estimates. Reference annotations should retain artery, vein, crossing or overlap, and uncertain classes. RITE illustrates this distinction by providing artery, vein, overlap, and uncertain labels rather than forcing all vessel pixels into only two classes (24).

Current methods take three main approaches to A/V labeling. Multi-task or dual-stream networks learn a shared vessel representation and produce separate artery and vein channels. Spatial embeddings and topology-ranking losses penalize implausible identity changes, whereas graph methods represent centerlines and junctions as nodes and edges before propagating labels through a minimum spanning tree or graph neural network. Girard et al. combined joint CNN segmentation with graph propagation and reported 94.8% vessel-segmentation accuracy, 93.7% sensitivity, and 92.9% specificity for A/V classification on CT-DRIVE. VTG-Net combined CNN and graph-convolution features and reported a mean A/V classification accuracy of 98.11% on AV-DRIVE under its evaluation protocol (25, 26). These findings support the use of global topology, although external validation and crossing-specific error analyses remain limited.

### 3.3 OCTA capillary networks: the segmentation input is already a processed product

OCTA measures flow-related decorrelation rather than vessel walls. Segmentation targets include the superficial and deep vascular plexuses, large vessels and capillaries, the foveal avascular zone (FAZ), non-perfusion areas, and macular neovascularization (MNV). Capillaries are dense and close to the resolution limit. Slow flow may disappear, noise may resemble flow, and the selected threshold can change apparent connectivity. Vessel density, skeleton density, FAZ area, and fractal dimension therefore describe the combined acquisition and processing pipeline rather than device-independent biological constants (10, 27–30).

An important source of error precedes vessel segmentation. Commercial en face slabs are generated after automated delineation of structural retinal boundaries. Macular edema, drusen, high myopia, discontinuous segmentation, and poor signal can displace those boundaries, causing a nominal superficial or deep slab to contain the wrong anatomy. Projection from superficial flow duplicates vessels in deeper slabs, while motion creates horizontal or vertical breaks. Clinical OCTA reviews report frequent motion, layer-misidentification, projection, and low-signal artifacts (28, 31). Projection-resolved and projection-artifact-removal methods can improve visualization and lesion measurement, but aggressive settings may also suppress true vertically connected flow (32).

OCTA segmentation studies should report the structural-layer software and version, any manual boundary correction, slab definitions, projection-artifact removal (PAR), signal-strength thresholds, motion correction, scan size, and criteria for excluding images with segmentation errors. In a deployable workflow, gradability and boundary plausibility should be assessed before vessel segmentation, and the upstream quality-control result should accompany every biomarker. Three-dimensional-to-two-dimensional networks such as IPN-V2 can learn projections directly from volumes and may reduce dependence on a fixed commercial slab, but anatomical localization still requires verification (33).

### 3.4 Structural OCT choroid, OCTA choriocapillaris, and MNV

Structural OCT analysis of the choroid and OCTA analysis of the choriocapillaris are separate tasks. In enhanced-depth or swept-source structural B-scans, the inner and outer choroidal boundaries are delineated and the cross-sectional tissue is divided into luminal and stromal compartments. The choroidal vascularity index (CVI) is the luminal area divided by the total choroidal area. This procedure segments tissue compartments rather than a connected vascular tree. Its main sources of error are an indistinct choroidoscleral interface, shadowing, scan-position dependence, and sensitivity to the binarization threshold (34–36).

OCTA choriocapillaris analysis instead identifies areas of reduced flow signal within a thin slab. Estimates of flow deficit depend on attenuation from the RPE or drusen, projection and compensation methods, slab depth, device sensitivity, interscan time, and the local or global threshold. Different thresholding methods can each be repeatable but still yield measurements that are not interchangeable, which makes direct pooling across studies unsafe (37). Unless an independent flow reference has been used, the appropriate label is ‘flow deficit’ rather than ‘nonperfused vessel.’

MNV/CNV is a related but distinct lesion-segmentation problem involving an irregular pathological vascular complex. En face OCTA can estimate lesion area, vessel density, junction density, and morphology, but the observed boundary varies with flow sensitivity and artifact removal (38). Bibliometric mapping describes research activity in AMD imaging rather than providing primary validation of an MNV segmentation model (39). Structural OCT activity features, fluorescein or indocyanine-green angiography, and clinical treatment decisions provide complementary evidence (40, 41). We suggest that evaluation of an MNV model include lesion-level detection, boundary agreement, longitudinal repeatability, and discrimination between quiescent and treatment-requiring disease, in addition to pixel Dice.

### 3.5 Corneal and conjunctival vasculature: segmentation versus redness grading

Anterior-segment vascular analysis comprises at least three task families. True vessel segmentation labels vessel pixels or centerlines and supports measurements of density, length per area, caliber, tortuosity, branching, and neovascular invasion. Region segmentation defines the cornea or exposed conjunctiva that serves as the denominator for those measurements. Hyperemia grading or redness indexing predicts a global color-based score and may correlate with Efron or McMonnies scales without reconstructing a vascular tree. The outputs are related, but a correlation between a redness index and a clinical grade does not establish vessel-segmentation accuracy (14, 42).

In corneal neovascularization (CoNV), slit-lamp photography projects vessels on a curved surface into two dimensions. Once magnification and the corneal region are standardized, these images can support estimates of visible area, length, density, and radial invasion. They cannot, however, determine true stromal depth. Depth-resolved assessment requires cross-sectional or volumetric imaging with AS-OCT or AS-OCTA, or localized *in vivo* confocal microscopy. AS-OCTA can combine en face maps with cross-sections, although its small field of view, shadowing from opacity, and sensitivity to motion remain important limitations. Any claim about vessel ‘depth’ should specify the imaging modality and coordinate definition (13, 43–45).

A slit-lamp study of 80 CoNV images trained IterNet on 60 images and tested it on 20. The model achieved an AUC of 0.989, accuracy of 0.988, sensitivity of 0.879, specificity of 0.993, and Dice of 0.879, with strong agreement in vessel-pixel count; deep vessels and those obscured by opacity were not visible (45). EVA used U-Net segmentation with connectivity-preserving analysis in 23 functional slit-lamp videos to estimate conjunctival microvascular structure and flow (46). These study-specific datasets support feasibility but do not establish multicenter generalizability or clinical benefit. Prospective multicenter cohorts need to represent variation in illumination, pigmentation, tear-film stability, eyelid or eyelash occlusion, topical medications, and repeated visits.

## 4 Vessel-specific architecture design

### 4.1 Geometry, class imbalance, and scale variance

Vascular segmentation differs from compact-organ segmentation because vessels have a high aspect ratio, occupy a small fraction of the image, branch across many scales, and may be only one or two pixels wide. A local break can disconnect an entire distal subtree while producing little change in global Dice. Architecture design must therefore preserve high-resolution detail and long-range context, adapt to caliber and curvature, and optimize connectivity as well as area.

Multiscale feature pyramids and U-Net skip connections combine semantic context with high-resolution features, but conventional downsampling can erase the thinnest vessels. Deep supervision at multiple scales, full-resolution branches, dilated convolutions, and coarse-to-fine or cascaded U-Nets can recover some of this detail. Deformable convolution learns sampling offsets that follow local curvature and caliber. DUNet reported accuracies of 0.9697, 0.9722, and 0.9724 and AUCs of 0.9856, 0.9868, and 0.9863 on DRIVE, STARE, and CHASE_DB1, respectively (47). Directional filters and oriented convolutions encode elongated structure and remain useful as fixed priors, auxiliary channels, or post-processing components when training data are limited.

Class-weighted cross-entropy, focal loss, Dice or Tversky objectives, hard-negative mining, and targeted patch sampling address foreground imbalance, but each can shift the balance between sensitivity and specificity. A supervised decision threshold or a threshold-selection rule should be fixed using development data before evaluation. Selecting a threshold to maximize agreement with test reference labels leaks outcome information. A prespecified adaptive rule that uses image intensities without test labels is not, by itself, evidence of data leakage. Accuracy is especially uninformative when background pixels dominate. In the Little W-Net study, a model with 68,482 parameters achieved results comparable to published larger architectures, but the literature comparison included differing data splits. A separate comparison with a 76,213-parameter U-Net used matched training conditions (48). This result suggests that disciplined data splitting, preprocessing, and evaluation can matter as much as architectural novelty.

### 4.2 Attention, transformers, state-space models, and diffusion

Attention modules reweight spatial locations or feature channels and can help when vessel contrast is uneven. Full self-attention at native ophthalmic resolution is memory intensive, so windowed and hierarchical Transformers trade some global context for lower computational cost. Their value should be tested against strong convolutional baselines trained under identical conditions. State-space sequence processing can scale linearly with sequence length, but this does not establish the memory use or end-to-end latency of a complete segmentation pipeline. Serp-Mamba was designed for high-resolution UWF-SLO images and combines serpentine scanning with local continuity cues to follow long vessel trajectories, giving a task-specific rationale for its backbone rather than treating state-space modeling as a generic replacement (49).

HiDiffSeg applies hierarchical diffusion to retinal fundus vessel segmentation (50). The relevant implementation trade-off is iterative sampling: repeated denoising steps can add computation, so latency should be measured for the actual sampling schedule, resolution, and hardware. This review does not establish a measured speed ranking against CNNs or Transformers. For stochastic segmentation, repeated samples can support an uncertainty analysis, but anatomical plausibility, calibration, topology, and biomarker agreement still require independent validation; visually detailed outputs alone do not establish accuracy.

### 4.3 Foundation models: adaptation is necessary but not sufficient

Retinal foundation models and general segmentation foundation models address different limitations. RETFound and FLAIR learn ophthalmic image representations from large retinal collections and can reduce the amount of labeled data needed for downstream disease tasks (51, 52). MedSAM and other SAM-derived methods provide promptable segmentation masks (53, 54). SAM-OCTA used low-rank adaptation and point prompts for OCTA vessels, FAZ, and A/V targets (55). Its author manuscript reports 10-fold cross-validation and an A100 GPU with 80 GB memory; the hardware specification is not a measured peak-memory requirement. vesselFM used synthetic domain randomization across three-dimensional vascular datasets to improve zero-shot transfer, although its broad vascular scope does not establish performance in every ophthalmic modality (56).

Promptable generalist models have predictable weaknesses in retinal microvasculature. A point or box prompt may include both vessel and background, low-contrast capillaries may be omitted, masks may follow lesion borders, and disconnected peripheral branches may be treated as separate objects. General medical evaluations of SAM have found wide variation across modalities and tasks, which supports domain-specific adaptation rather than an assumption of reliable zero-shot performance (57). Fine-tuning only the decoder or low-rank adapters reduces the number of trainable parameters but does not resolve pretraining bias, ambiguous annotations, or topological error. In the author manuscript of SAM-OCTA, prompts were sampled using reference-mask components and background (55). Such simulated prompts are not equivalent to independent clinician prompts or autonomous inference; comparisons must account for prompt information and selection of prompt settings.

A rigorous foundation-model study should identify the checkpoint and prompt protocol, disclose training images and possible overlap with the benchmark, and state which components were frozen or trained. It should also report parameter count, GPU memory, inference time, sensitivity to prompt number and location, external cross-device performance, and errors involving leakage, missed terminal vessels, and crossings. Prompt design must be fixed before test evaluation. Supervised fine-tuning, self-training, feature alignment, style augmentation, and test-time adaptation can improve domain fit, but pseudo-labeling may reinforce missed capillaries. Human correction is informative only when correction time and interobserver variability are measured.

## 5 Evaluation from pixels to clinical action

No single metric captures vascular segmentation quality. Table 1 separates five complementary levels: pixel overlap, centerline recovery, topology/graph structure, biomarker agreement, and clinical actionability. This is an organizational framework for critical synthesis, not a validated composite score. Pixel metrics assess local classification; centerline metrics emphasize thin-vessel localization; graph metrics assess connectivity and identity; biomarker metrics test the derived measurement; and clinical metrics examine decision relevance and workflow effects. Good results at one level do not guarantee acceptable performance at the next.

**Table 1:** A five-level evaluation framework for ophthalmic vascular segmentation.

Level	Representative metrics	What it measures	Key limitation
Pixel	Dice, IoU, sensitivity, precision, PR-AUC	Local foreground/background agreement	Background-dominated accuracy; weak sensitivity to a single broken branch
Centerline	clDice, centerline distance, length error	Recovery and localization of thin vessels	Depends on skeletonization; may underrepresent caliber
Topology/graph	*β*0/β1 error, junction F1, component count, graph edit/matching	Connectivity, loops, branch structure, identity continuity	Reference graph extraction and crossing definitions vary
Biomarker	Bias/limits of agreement, ICC, repeatability of VD, FAZ, caliber, tortuosity	Stability of the intended clinical measurement	A stable biased mask can still yield a biased biomarker
Clinical	AUROC at patient level, calibration, longitudinal change, correction time, outcome association	Decision relevance and workflow effect	Requires prospective, representative cohorts

FAZ, foveal avascular zone; ICC, intraclass correlation coefficient; IoU, intersection over union; PR-AUC, area under the precision-recall curve; VD, vessel density.

Dice is 2TP/(2TP + FP + FN), and IoU is TP/(TP + FP + FN). Both vary with vessel prevalence and caliber. Sensitivity captures missed vessel pixels, whereas specificity is dominated by background; AUC can also hide performance at the threshold selected for clinical use. Precision-recall curves are more informative under severe class imbalance. Studies that propose a quality-control threshold or a trigger for human review should additionally report calibration and uncertainty.

Structural evaluation can include centerline distance for localization after skeletonization, junction precision and recall for branching, connected-component error for fragmentation, and graph-edit or matching distance for node-edge agreement. clDice combines skeleton-to-mask overlap in both directions; a soft-skeleton formulation provides a differentiable training loss for tubular structures (58). Persistent-homology methods track the appearance and disappearance of connected components and loops across thresholds. In two dimensions, β0 counts connected components and β1 counts loops. Topological losses can penalize discrepancies from a specified reference topology even when pixel overlap changes little (59). Normal capillary loops must not be penalized simply because they violate a tree prior. At capillary scale, however, both skeletonization and manual annotation can be unstable, so structural metrics are best interpreted together.

Biomarker validation should compare automated and reference measurements using bias, limits of agreement, intraclass correlation, coefficient of variation, and repeatability across visits and devices. In an OCTA benchmark, Giarratano et al. reported a Dice of 0.89 for U-Net/CS-Net but found differences of up to 25% in vessel-density accuracy across segmentation methods (60). This discrepancy shows why overlap alone cannot establish biomarker validity. Clinical actionability requires prespecified outcomes, including disease discrimination, progression prediction, association with treatment response, clinician correction time, and evidence that the system changes management without increasing unsafe errors.

## 6 Datasets and quantitative benchmarking

Tables 2–4 summarize fundus, OCTA, and anterior-segment resources, respectively; Table 5 presents representative reported metrics together with validation and implementation context. These are descriptive comparisons, not a common-protocol ranking. Public fundus datasets support controlled development but are often small. OCTA evidence also depends on slab definition, scan field, and annotation type. Among the sources considered here, anterior-segment segmentation is supported mainly by study-specific resources rather than a widely adopted public benchmark with standardized vessel labels.

**Table 2:** Representative retinal fundus, SLO, and UWF vascular datasets.

Dataset	Size	Image source/acquisition	Annotation target	Main caveat
DRIVE	40 (20/20)	Color FP; 565 × 584; 45°	Binary vessel masks	Small fixed split; DR-screening cohort
STARE	20	Color FP; 700 × 605; ∼35°	Binary vessel masks	Very small; mixed pathology
CHASE_DB1	28 images/14 children	Color FP; 999 × 960; 30°	Binary vessel masks	Pediatric-only; eye-level dependence
HRF	45	Color FP; 3504 × 2336; 60°	Binary vessel masks	15 healthy, 15 DR, 15 glaucoma
DR HAGIS	40	Color FP; multi-device; 45°	Binary vessel masks	Disease-focused; uneven resolution
IOSTAR	30	SLO; 1024 × 1024; 45°	Vessels; associated A/V labels	Different contrast and artifacts from flash FP
RITE	40 (DRIVE-derived)	Color FP; DRIVE images	Artery, vein, crossing, uncertain	Shares images/splits with DRIVE (24)
LES-AV	22	Color FP; 30°/45°; disc-centered	A/V and vessel labels	11 healthy, 11 glaucoma; variable illumination
INSPIRE-AVR	40	Color FP	Expert-averaged AVR reference	Two-expert IVAN assessment; not a pixel-mask benchmark (61)
PRIME-FP20	15 paired UWF FP/FA images	UWF SLO-based FP/FA; ∼200°	*De novo* vessel reference for evaluation	Weak-supervision benchmark; peripheral distortion (23)

Dataset characteristics refer to the releases described in the cited sources; later releases and annotation extensions may differ (23, 24, 48, 61).

**Table 3:** Public OCTA datasets with acquisition and annotation details.

Dataset	Size/scan field	Device/sampling	Available targets	Main caveat
OCTA-500	500 subjects: 300 6 × 6 mm; 200 3 × 3 mm	RTVue-XR Avanti, Optovue; 70-kHz SD-OCT	OCT/OCTA volumes; 6 projections; large vessel, capillary, artery, vein, 2D/3D FAZ, retinal layers	Disease distribution differs between subsets (33)
ROSE-1	117 images/39 subjects; 3 × 3 mm	RTVue XR Avanti, Optovue; 304 × 304	SVC and SVC + DVC: pixel/centerline labels; DVC: centerline only	Repeated slabs per subject; small test set (48, 62)
ROSE-2	112 images; 3 × 3 mm	Spectralis OCT2, Heidelberg; 512 × 512 A-scans; images resized to 840 × 840	SVC; centerline vessel labels	Different device/appearance; no pixel-width label (48, 62)
Giarratano OCTA benchmark	55 macular ROIs/11 subjects without ocular disease; 3 × 3 mm	RTVue XR Avanti, Optovue; 91 × 91 ROIs	Manual capillary segmentation; vascular metrics	Foveal+parafoveal ROIs; subject-level split not established from report (60)

DVC, deep vascular complex; FAZ, foveal avascular zone; OCTA, optical coherence tomography angiography; ROI, region of interest; SD-OCT, spectral-domain OCT; SVC, superficial vascular complex.

**Table 4:** Representative anterior-segment and ocular-surface vascular cohorts.

Study/resource	Size	Acquisition	Target/reference	Availability/limitation
Chu et al., CoNV	80 images; 60 train/20 test	Topcon SL-D701 slit lamp; 10×; 3264 × 2448	Visible CoNV pixel mask; vessel-pixel count	Private, single center; 2D only (45)
EVA conjunctival videos	23 videos; 30 vessel segments for velocity	Functional slit-lamp microscopy	Vessel centerlines, segment length, blood velocity	Private; hemodynamic protocol (46)
CVNet	Study-specific cohort	Custom optical imaging; vessel-guided Raman positioning	Conjunctival vessel mask	Study-specific imaging system (63)
Dry-eye microvasculature	51 patients: 17 SSDE/34 NSDE	Custom optical video system; motion correction; Attention-UNet	Diameter; blood flow velocity and rate	Prospective recruitment; cross-sectional, single-center analysis (64)
Semisupervised hyperemia	317 primary-site + 164 external-site images	Slit-lamp photography	Conjunctiva + vessel masks; Efron grade	Two-site retrospective; segmentation and Efron correlation are distinct (42)

The listed anterior-segment resources are study-specific; segmentation accuracy, hemodynamic association, and global hyperemia grading are distinct endpoints*.*

**Table 5:** Representative reported segmentation results; values are not directly rankable across rows.

Model	Dataset/task	Metric	Reported value	Interpretive note
DUNet	DRIVE/STARE/CHASE_DB1	Accuracy; AUC	0.9697/0.9722/0.9724; 0.9856/0.9868/0.9863	Deformable U-Net; within-dataset protocols (47)
Little W-Net	DRIVE/CHASE_DB1/HRF	Dice; AUC; MCC	0.8279/0.8169/0.8103; 0.9810/0.9847/0.9825; 0.8024/0.7974/0.7909	68,482 parameters; five-run means; within-dataset evaluation (48)
Little W-Net cross-dataset	Train DRIVE → UoA-DR	Dice; AUC; MCC	0.3829; 0.8232; 0.3551	Single trained model; source DRIVE Dice 0.8282; no target retraining (48)
OCTA-Net	ROSE-1 SVC/SVC + DVC/DVC; ROSE-2	Dice; AUC	0.7697/0.7576/0.7074/0.7077; 0.9453/0.9375/0.9673/0.8603	Mixed pixel-width/centerline references; values reported in Tables 6 and 7 of reference 48 (48, 62)
Little W-Net	ROSE-1 SVC/SVC + DVC/DVC; ROSE-2	Dice; AUC	0.7903/0.7847/0.7082/0.6970; 0.9495/0.9405/0.9771/0.8625	Mixed label types; within-dataset training, not cross-device transfer (48)
U-Net/CS-Net	Giarratano OCTA benchmark	Dice	0.89 each	30 train/25 test ROIs; patient-level separation not established (60)
CNN + graph propagation	CT-DRIVE A/V	Vessel accuracy; A/V sensitivity; specificity	0.948; 0.937; 0.929	Graph propagation after joint CNN (25)
IterNet	CoNV slit-lamp test set (*n* = 20)	Dice; AUC; sensitivity; specificity	0.879; 0.989; 0.879; 0.993	60 train/20 test images; Tesla P100 16 GB training hardware (45)
SAM-OCTA (preprint v1)	OCTA-500 RV; 3 × 3/6 × 6 mm	Dice	0.9199/0.8869	10-fold CV; ViT-H + LoRA; simulated prompts; A100 80 GB (55)

Values are transcribed from the cited reports, not pooled or independently recomputed. Dataset order follows the task column. Different splits, reference labels, thresholds, and prompting conditions prevent direct ranking. GPU specifications describe reported training hardware, not peak memory or deployment latency. SAM-OCTA values refer to the preprint version associated with reference 55 (arXiv:2309.11758v1, 2023), not a separate later article with a similar name; the IEEE final text has not been independently checked here.

## 7 Biomarker validity, ocular biometry, and cross-domain robustness

### 7.1 Magnification correction and physical units

Caliber, length, and area measurements require a calibrated lateral image scale to be expressed in physical units. Dimensionless measures such as area fraction or AVR need clearly defined regions and formulas; their sampling regions can still be affected by magnification. Fundus cameras and OCT systems commonly assume a model-eye axial length, although axial length, refractive error, and keratometry all affect ocular magnification. Without correction, the same anatomical structure can occupy a different number of pixels, biasing measurements of caliber, area, length density, FAZ area, and the choriocapillaris. Sampson et al. showed that axial-length variation changes estimates of superficial retinal vessel density and FAZ area on OCTA (65).

Littmann-type scaling expresses actual retinal size as the product of an instrument factor and an ocular factor. Bennett's approximation for the axial-length component is often written *q* = 0.01306(AL − 1.82), where AL is axial length in millimeters and instrument-specific calibration completes the scale (65). Axial length is preferred when available. Refractive error and keratometry can supplement model-eye calculations but cannot replace device calibration. Studies should state whether scaling was applied before or after segmentation, report the formula and assumed model eye, and provide results in both pixels and physical units. Otherwise, a model may learn an association with magnification rather than disease.

### 7.2 Cross-device and cross-dataset testing

Domain shift can arise from camera spectrum, resolution, field of view, compression, population pigmentation, disease mix, scan protocol, slab algorithm, or annotation style. In the Little W-Net analysis, the single-model cross-dataset experiment reported a Dice of 0.8282 on DRIVE and 0.3829 on UoA-DR without target-dataset retraining, with variable losses across nine external datasets (48). A high internal AUC gives no warning of deterioration on this scale. Cross-domain results should identify the training site and device, preserve an untouched external test set, report disease and image-quality strata, and calculate confidence intervals at the patient level rather than the image-patch level.

Proposed responses to domain shift include color or style augmentation, spatial resampling, histogram standardization, domain-adversarial training, self-training, source-free or test-time adaptation, and federated learning. For offline adaptation, comparison of a locked source model, a prespecified adaptation procedure, and a target-supervised reference should use an independent target test cohort and disclose the target-label budget. Test-time or transductive adaptation may access unlabeled test images under a declared protocol; this setting must be distinguished from evaluation of a model with no target-test access. None is risk free. Style transfer may alter lesions, resampling changes apparent caliber, pseudo-labels can reinforce missed vessels, test-time adaptation may drift on a small batch, and federated sites may still share the same device bias. A locked, non-adaptive model provides a regulatory baseline. Adaptation after deployment should be versioned, monitored, and assessed under the applicable regulatory change process; where an authorized change-control plan is used, modifications must remain within its scope.

## 8 From algorithm to clinical workflow

### 8.1 Intended use and prospective evidence

Table 6 summarizes a proposed deployment checklist, not evidence that these steps have already been completed for the reviewed models. Clinical development begins with a precise intended-use statement covering the target population, modality, acquisition protocol, output, user, setting, and resulting action. Software that exports vessel masks for retrospective research carries a different risk from a system that changes referral, treatment, or follow-up. Evidence should progress from technical validation to external retrospective validation, silent prospective deployment, reader or workflow studies, and outcome or management-impact evaluation. Prospective protocols should define how failures will be handled, prespecify subgroup analyses, measure correction time, and determine whether biomarker changes exceed test-retest variability (66, 67). As an example of clinical association evidence, Lee et al. prospectively recruited 51 patients with dry eye and used motion-corrected conjunctival imaging with Attention-UNet segmentation to compare vessel diameter and flow measures (64). The analysis was cross-sectional and single-center; it did not test whether AI-guided management improves patient outcomes. Prospective recruitment should therefore be distinguished from a prospective clinical-impact trial.

**Table 6:** Translation-readiness checklist for ophthalmic vessel AI.

Layer	Minimum evidence	Operational control
Acquisition	Supported devices, scan/FOV, ocular biometry, gradability	Reject unsupported input; record raw image and metadata
Anatomical preprocessing	Layer boundaries, PAR, corneal/conjunctival ROI	Show QC overlays; retain corrected and original versions
Segmentation	External Dice + topology + subgroup errors	Uncertainty and post-segmentation plausibility checks
Biomarker	Scale and ROI definition, repeatability, bias/limits of agreement	Versioned formula; validated reference range if available
Workflow	DICOM/PACS routing, latency, edit time, alert burden	Human confirmation and audit log
Clinical evidence	Prospective design, management impact, safety endpoints	Monitor drift, complaints, and subgroup performance
Regulation	Intended use, risk class, cybersecurity, change plan	Locked release or controlled, validated updates

FOV, field of view; OOD, out-of-distribution; PACS, picture archiving and communication system; PAR, projection-artifact removal; ROI, region of interest.

### 8.2 SaMD regulation and model change

Segmentation software that informs diagnosis or treatment may be regulated as software as a medical device (SaMD). In the United States, possible marketing routes include 510(k) clearance based on substantial equivalence, *de novo* classification for a novel low- or moderate-risk device without a legally marketed predicate, and premarket approval for higher-risk uses. The route depends on intended use and risk, not on the use of the term AI. The FDA's public list of AI-enabled devices confirms that authorization applies to a specific device and indication (68). A predetermined change control plan may describe planned model modifications, their validation, and impact assessment; it does not permit unconstrained continuous learning (69).

In the European Union, the Medical Device Regulation (EU) 2017/745 governs medical-device software, while related Medical Device Coordination Group guidance supports interpretation of qualification and classification (70, 71). The evidence package should address clinical evaluation, risk management, cybersecurity, human factors, data governance, post-market surveillance, and traceability from model version to output. A single regulatory class cannot be assigned to all vessel-segmentation tools. Classification depends on the claimed clinical purpose and the consequences of an incorrect result.

### 8.3 PACS/DICOM integration, OOD quality control, and auditability

Clinical integration requires more than a dashboard. Ophthalmic images and derived objects must remain linked to the patient, study, series, laterality, device, scan protocol, and processing metadata in the PACS or vendor archive. DICOM Supplement 197 defined storage classes for ophthalmic OCT en face images and B-scan volume analysis (72). Device-specific export and PACS import should be verified against the relevant conformance statements; the existence of a standard does not establish implementation in a particular product. If native DICOM is unavailable, the system should retain the original files and record provenance explicitly instead of relying on converted screenshots.

Out-of-distribution (OOD) and quality-control checks are needed before and after segmentation. Input checks should verify modality and laterality, field of view and resolution, signal strength, focus, illumination, motion, layer-boundary plausibility, and the presence of unsupported pathology. Output checks should assess vessel fraction, connected components, terminal-branch loss, loops inconsistent with the modality-specific reference anatomy, artery-vein conflicts, FAZ plausibility, and uncertainty. Images outside the validated range should be rejected or sent for human review. Each displayed result should record the confidence or failure reason, model and preprocessing versions, manual edits, and final biomarker provenance.

Explanations should correspond to the segmentation task. Saliency maps are often too coarse to account for a pixel-level mask. More informative displays mark uncertain segments, show the structural slab used to generate an OCTA projection, compare images before and after PAR, identify branches that drive a biomarker change, and quantify the effect of manual correction. Computational reports should state the number of trainable parameters, hardware, peak memory, input size, inference latency, and any user time needed for preprocessing or prompting. These details determine whether the system can operate near the imaging device, on a workstation, or only in the cloud.

## 9 Discussion: critical synthesis and research priorities

The literature supports several consistent conclusions. Architectural complexity alone does not predict clinical usefulness: compact networks can match larger models on curated benchmarks, and both can fail after a shift in device, population, or pathology. Important gaps between a vessel mask and clinical use include unmeasured topological and biomarker error, limited external validation, and a lack of demonstrated workflow benefit. The modalities can share a validation framework, but their tasks remain different. Fundus and OCTA segmentation delineates vascular networks; tree priors are useful for large retinal arteries and veins, whereas capillary networks may contain normal loops; structural choroid analysis partitions tissue; choriocapillaris analysis maps flow deficits; MNV analysis segments a lesion; and hyperemia grading predicts a global phenotype.

A useful benchmark would include a locked multicenter test set, patient-level separation, device and disease strata, raw images with acquisition metadata, explicit ungradable cases, and reference labels that preserve uncertainty and crossings. Evaluation should combine pixel metrics with clDice or centerline distance, component and junction errors, and agreement for prespecified biomarkers. OCTA datasets should also provide structural boundaries or boundary-quality labels and document PAR. Fundus and UWF datasets should record physical scale and peripheral geometry. Anterior-segment datasets should distinguish two-dimensional visible-vessel outcomes from depth-resolved AS-OCT or AS-OCTA outcomes.

The most informative next studies are prospective comparisons conducted under a common protocol. Priorities include head-to-head testing of compact CNN, Transformer, Mamba, diffusion, and adapted foundation-model baselines; external evaluation across devices and sites without threshold retuning; and controlled analyses of lesions and artifacts. Studies should also compare automated and manually corrected biomarkers using limits of agreement and test-retest repeatability, measure correction time and management changes in reader studies, and evaluate OOD rejection during post-deployment monitoring. Foundation models should be judged by annotation savings and cross-domain robustness after accounting for computation and prompting, not by parameter count or the visual appeal of selected masks.

A clinical report should identify the source image and slab, display quality-control status and the segmentation overlay, and provide the physically corrected biomarker value with a validated reference interval, where available, or a longitudinal comparison under a matched acquisition protocol. It should also show uncertainty or failure flags and preserve a record of manual edits. With this information, a clinician can judge whether an apparent vascular change is biological, acquisition related, or algorithmic. A standalone probability map does not provide that level of auditability.

## 10 Limitations of this review

This structured narrative review was designed to compare modalities, task definitions, and translation requirements; it was not a systematic review or meta-analysis. Selection was purposive rather than exhaustive, so selection and publication bias remain possible; incomplete access to source reports can further limit appraisal. We did not enumerate every architecture, rerun algorithms, or reanalyze metrics from raw predictions. Values from studies that used different data splits, thresholds, disease spectra, or annotations are not directly comparable. Public information is also limited for proprietary preprocessing, computational cost, and unsuccessful external validation. Because research on foundation and state-space models is developing quickly, the newer methods discussed here will need continued reassessment.

## 11 Conclusions

Ophthalmic vessel segmentation now includes deep, topology-aware, and general-purpose models in addition to handcrafted enhancement methods. Clinical translation depends on the performance of the entire measurement pipeline. A useful system must preserve anatomy, produce stable biomarkers with appropriate units and definitions, identify unsupported inputs, and improve a defined clinical workflow; a plausible mask on a familiar benchmark is insufficient.

Fundus vessel segmentation has the longest benchmark history, yet performance can fall sharply across datasets. OCTA provides capillary and layer-specific information but inherits errors from structural-layer segmentation and projection artifacts. Structural OCT CVI, OCTA choriocapillaris flow deficits, and MNV lesions require distinct terminology and validation. Corneal and conjunctival vessel analysis has potential clinical value but needs shared datasets and depth-aware task definitions. Progress should be assessed at the pixel, centerline, topology, biomarker, and clinical levels, with prospective validation, interoperability, regulatory planning, and human oversight incorporated into study design.
